# An Extended Methodology for Sizing Solar Unmanned Aerial Vehicles: Theory and Development of a Python Framework for Design Assist

**DOI:** 10.3390/s21227541

**Published:** 2021-11-12

**Authors:** José Roberto Cândido da Silva, Gefeson Mendes Pacheco

**Affiliations:** Department of Microwave and Optoelectronics, Aeronautics Institute of Technology—ITA, São José dos Campos 12228-900, Brazil; gpacheco@ita.br

**Keywords:** photovoltaic generators, solar cell, solar unmanned aerial vehicles, Python, aircraft design

## Abstract

There is a growing interest in using unmanned aerial vehicles (UAVs) in the most diverse application areas from agriculture to remote sensing, that determine the need to project and define mission profiles of the UAVs. In addition, solar photovoltaic energy increases the flight autonomy of this type of aircraft, forming the term Solar UAV. This study proposes an extended methodology for sizing Solar UAVs that take off from a runway. This methodology considers mission parameters such as operating location, altitude, flight speed, flight endurance, and payload to sizing the aircraft parameters, such as wingspan, area of embedded solar cells panels, runway length required for takeoff and landing, battery weight, and the total weight of the aircraft. Using the Python language, we developed a framework to apply the proposed methodology and assist in designing a Solar UAV. With this framework, it was possible to perform a sensitivity analysis of design parameters and constraints. Finally, we performed a simulation of a mission, checking the output parameters.

## 1. Introduction

The energy generated by the sun, both as a source of heat and light, is today one of the most promising alternatives to meet the global energy demand. Among the various solar energy applications, we can mention solar power plants, commercial and residential use, transport, rural electrification, solar roads, satellites, and unmanned vehicles.

Unmanned vehicles are motor vehicles that do not have a human operator, are operated autonomously or remotely. These vehicles can carry various loads depending on their type, functionality, characteristics, and mission objectives and profiles [1].

These types of vehicles are classified as unmanned ground vehicles, unmanned underwater vehicles, unmanned surface vehicles (operating on the water’s surface), unmanned spacecraft, and unmanned aerial vehicles. Unmanned aerial vehicles (UAVs) present several advantages compared with manned aircraft, mainly in relation to human restrictions. Thus, for missions that can expose the pilot to a high workload or risks, the UAV is highly recommended.

The number of application areas where UAVs can become useful is almost unlimited, and range from military to daily civilian tasks, such as precision agriculture, communications, search and rescue, inspection of facilities and infrastructure, atmospheric satellites, environmental studies, mapping, and continuous border surveillance [1].

These applications have been the subject of interest in several types of research in recent years. In [2,3,4,5], extensive research discusses multiple UAV applications, such as remote sensing (RS), search and rescue (SAR), precision agriculture (PA), infrastructure inspection, road traffic monitoring, and delivery of goods.

Conventional terrestrial communication infrastructure deployment is not workable in some practical scenarios, for example, in dangerous spots, complex terrains, or disaster areas. On the other hand, UAVs equipped with built-in wireless transceivers can be an alternative communication infrastructure because of their high maneuverability and coverage capability [6]. In [7,8,9,10,11], the authors present a detailed survey about UAVs’ contribution, benefits, and challenges in wireless communications. In [12,13], the authors present a detailed survey about the use of 5G technology in communication networks based on UAVs.

In communications, satellites are widely used in image capturing missions. However, launching a satellite is not an easy feat, as it requires up-to-date technology and a tremendous investment in time and money. In addition, there is a recent discussion about the growing amount of space debris caused by satellites out of operation in Earth’s orbit [14]. For image capturing missions in regions of limited size, the use of a UAV requires less design and implementation time, having a better cost-benefit ratio. The research in [15] compares remote sensing in satellites and UAVs [16].

Using unmanned aerial vehicles in traffic monitoring applications is becoming more and more popular. Compared with traditional monitoring devices, UAVs are more cost-effective. In addition, traditional traffic monitoring devices capture traffic conditions at fixed locations; hence, it requires many units to monitor a single road segment. In contrast, a few UAVs can cover a continuous stretch of roadway or even a traffic network.

In natural disasters such as floods, tsunamis, and earthquakes or man-made disasters, such as terrorist attacks, telecommunications systems can be partially or entirely affected. Such situations require rapid solutions to provide communications coverage to support rescue operations [17]. UAVs can provide timely disaster warnings and help speed up rescue and recovery operations when the public communication network is disrupted. In [18], new research was developed using UAVs with pattern recognition and the ability to provide real-time images and advice, for example during forest fires, to the firefighters. Besides that, the UAV can have a router that enables the firefighters to communicate via email, text messaging, and voice mail when the fire destroys the communication infrastructure [18].

There is a growing interest in UAV uses in extensive construction projects monitoring [19] and autonomous navigation for high voltage power lines inspection [20,21,22,23], oil and gas pipelines, wind turbines, and GSM towers infrastructure inspection [17,24].

UAVs can transport food, packages, and other goods [25]. In addition, ambulance drones can deliver medicines, vaccines, and blood samples into and out of unreachable places in the healthcare field. With the rapid demise of snail mail and the massive growth of e-commerce, postal companies have been forced to find new methods to expand beyond their traditional mail delivery business models. Therefore, different postal companies have undertaken various UAV trials to test the feasibility and profitability of UAV delivery services [26].

The mentioned applications require the aircraft to perform long-endurance flights. However, the capabilities of energy sources limit this type of flight. Such constraint has been a subject of interest in various research worldwide, aiming to adopt alternative forms of energy, such as photovoltaic solar energy, giving rise to the term Solar UAV. Some areas of research have been prominent in Solar UAVs, such as improve energy consumption [27] and recharge methods [28], and the development of more efficient components [29]. Solar power as an energy source allows Solar UAVs to perform long-endurance flights, or even perpetual flight, at almost zero emission. Furthermore, Solar UAV is highly efficient because refueling is not required [14].

The operating principle of a Solar UAV is to capture the energy of solar radiation and transform it into electrical energy to supply the propulsive system, actuators, and embedded systems. The surplus electrical energy is stored in a block to supply energy demand when there is no solar radiation, such as on very cloudy days or even in night operations.

The area of the solar cells, from which the energy needed for the aircraft’s flight comes, impacts the wing area and the total weight of the aircraft, which in turn affects the amount of energy needed.

Understanding and improving the design steps of this type of critical system represents a significant technological advance. Not only for solar-powered UAVs but also for all photovoltaic systems, which can significantly increase overall efficiency and improve its cost-benefit.

Previous studies related to Solar UAVs [30,31,32,33] have performed a proof-of-concept of this type of aircraft. The state-of-the-art solar-powered perpetual flight of low-altitude aircraft was achieved in the UAV developed in [30], based on studies in [31,32]. This UAV achieved a flight of 81 h and 2338 km ground distance. In addition, based on [32,33], the author in [30] developed a Matlab-based conceptual design and analysis framework for solar-powered UAVs. 

In [34], the authors proposed a methodology for the conceptual design of Solar UAV, based on [32], and carried out intensive research to verify the project sensitivity for some mission parameters changes from simulations performed on a framework developed in Matlab. The work in [35] applied the methodology proposed in [34], suggesting some simplifications in the input parameters, such as the use of commercially available solar cells and storage blocks. After performing simulations with Solar UAVs, the methodology accuracy was verified using previous authors’ aircraft data, with consistent results. A new Matlab framework and a ground model of a Solar UAV were built to validate the methodology proposed in [34].

Recent work in Solar UAVs [36,37] has shown interest in developing conceptual design algorithms, with the possibility of parameter optimization. In [38] the authors proposed an optimization solution using genetic algorithms.

Therefore, this work aims to understand the main parameters in the design of a low altitude long endurance (LALE) Solar UAV, based on [30,31,32], and to extend these methodologies beyond the early contributions proposed in [34,35]. An extended model for sizing Solar UAVs that takeoff and land on a runway, operate in variable endurance, and assemble with commercially available components is proposed. One of the main objectives of the proposed methodology is to simplify the number of parameters provided by the user. Furthermore, this methodology aims to build a Solar UAV with an automatic operating cycle, one that can take off automatically when the batteries reach full charge, necessary for takeoff, cruise flight, and landing. When the battery charge drops to a safety limit, due to the low incidence of solar radiation, the aircraft makes a landing to recharge the batteries. Thus, a new mission cycle begins. Therefore, a detailed study of the energy supply responsible for the aircraft’s takeoff and landing is necessary, as well as its impact on the aircraft sizing. The main objectives of this study are summarized as follows:

- Develop a complete model for estimating solar radiation on the day of the year with less incidence of sunlight, with no mission site-specific weather data provided by weather stations. The user only needs to inform the operating location, and the algorithm estimates incident solar radiation at that location;
- Simplify aerodynamic data input from the model, using an aerodynamic profile database, containing the various aerodynamic parameters necessary for the aircraft sizing;
- Develop a model to estimate the power needed for the flight, including the different flight phases (takeoff, climb, cruise descent, and landing). This model must find the optimal relationship between the aircraft’s total weight and the power needed to maintain the cruise flight since the highest value of the mass/power ratio is desirable. Such parameters are close to the related mission profile;
- Develop a model to estimate the power needed for the flight, including the different flight phases (takeoff, climb, cruise descent, and landing). This model must find the optimal relationship between the total weight of the aircraft and the power needed to carry out the cruise flight since the highest value of the mass/power ratio is desirable.
- Develop a free open-source framework, with Python language, that is easy to use and that allows the definition of the aircraft route, besides performing aircraft sizing, detailed analysis of design constraints and aircraft optimization.

## 2. Solar Unmanned Aerial Vehicles

The operating requirements of the mission determine the characteristics of the UAV, such as shape, size, performance and costs. In addition, other parameters, such as geographic position, aerodynamics, photovoltaic and propulsion characteristics, directly impact the aircraft. From these parameters, the designer creates an initial design of the aircraft as a starting point for a more detailed project or as the first project of iteration [34].

### 2.1. Aerodynamic and Fuselage Settings

The aerodynamic theory used for UAVs is the same as that used for the conceptual design of conventional aircraft [39]. We do not explore the aerodynamic theories deeply, just enough to develop the project focusing on the sizing of the photovoltaic system.

Aircraft wings can take various geometric shapes according to the purpose of the design, but the main types are rectangular, trapezoidal, elliptical, and mixed. Each one has its particular characteristic with advantages and disadvantages when compared with each other.

The rectangular wing has a low aerodynamic efficiency compared with a trapezoidal or elliptical wing. Its advantage is its greater ease of construction and lower manufacturing cost compared with others. In addition, the rectangular shape makes the assembly of solar cells easier.

The planar area of a rectangular wing can be calculated from:

(1) S=b·c,

where *b* is the wingspan and c is the profile chord.

The aspect ratio (AR) represents the ratio between wingspan and profile chord, as shown in the following equation [40]:

(2) $$AR=\frac{b}{c}.$$

**Figure 1:**
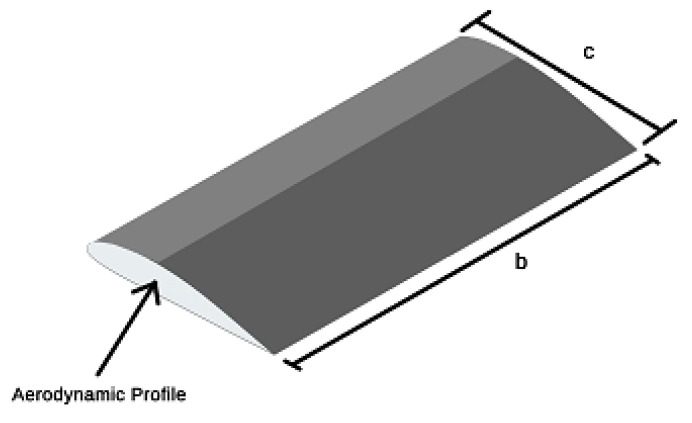
Main features of a rectangular wing.

Figure 1 shows a rectangular wing and its main geometric features, including its aerodynamic profile, defined by the wing surface in order to obtain an aerodynamic reaction from the flow of the surrounding fluid.

Each aerodynamic profile has its aerodynamic characteristics, which depend only on its geometric shape, dimensions, bending, thickness, and leading-edge radius. The main aerodynamic characteristics of a profile are the lift, drag, and moment coefficient, the position of the aerodynamic center, and its aerodynamic efficiency [40].

**Figure 2:**
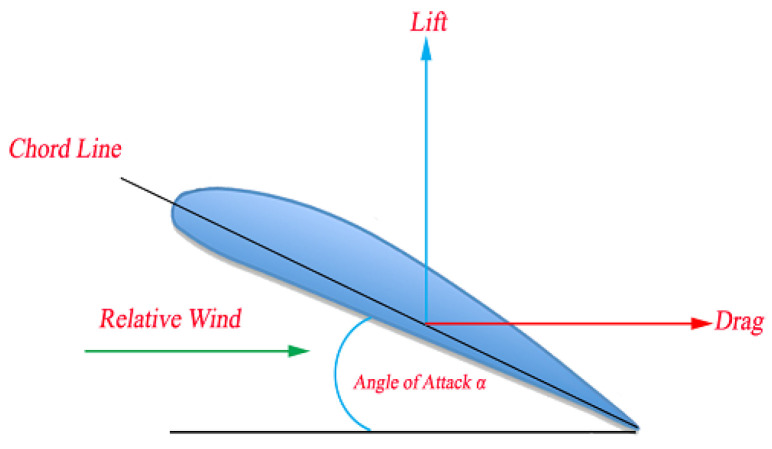
Definition of the angle of attack of the aerodynamic profile.

The angle of attack α is the term used by aerodynamics to define the angle formed between the profile line chord and the relative wind direction. It represents a parameter that decisively influences the ability to generate profile lift. Usually, the increased angle of attack increases the lift force up to a certain point at which it abruptly decreases. This point is a stall. Increasing the angle of attack also allows the drag to be generated. The dependence of the lift and drag with the angle of attack can be measured by the lift and drag coefficients. Figure 2 shows an aerodynamic profile and its respective angle of attack.

The lift coefficient represents the efficiency of the profile in generating the lift force, usually determined from a wind tunnel or specific software simulating a wind tunnel. The lift coefficient is a function of the profile model, the Reynolds number, and the angle of attack.

The drag coefficient represents how much drag force the aerodynamic profile produces. The aerodynamic profile is aerodynamically efficient when producing large lift coefficients and small drag coefficients. For an aerodynamic profile, the drag coefficient is also a function of the Reynolds number and the angle of attack.

The forces acting on a wing can be calculated by applying the following equations [40]:

(3) $$L=\frac{1}{2}\;\cdot \rho \cdot v^{2}\cdot S\cdot C_{L},$$

(4) $$D=\frac{1}{2}\;\cdot \rho \cdot v^{2}\cdot S\cdot C_{D},$$

where L represents the lift force, D represents the drag force, ρ represents the air density, v is the velocity of the flow, S is the wing area, $C_{D}$ is the drag coefficient and $C_{L}$ is the lift coefficient.

For a wing of finite dimensions, the drag coefficient $C_{D}$ is the sum of the profile drag $C_{D0}$ with the induced drag $C_{Di}$, generated by the wing-tip vortices [40]:

(5) $$C_{D}=C_{D0}+C_{Di}.$$

The profile drag $C_{D0}$ is experimental and contains all the complex dependencies of the aerodynamic profile.

Mathematically, for a wing with AR ≥ 4, we define the induced drag as [40]:

(6) $$C_{Di}=\frac{C_{L}^{2}}{\pi \cdot e\cdot AR},$$

where e is the wingspan efficiency factor or Oswald efficiency factor. This factor represents a parameter that depends on the geometric model and aspect ratio of the wing. The Oswald efficiency factor can be estimated by [41]:

(7) $$e=\frac{1}{Q+P\pi AR},$$

where Q contains the inviscid part of the induced drag coefficient, and the term P is used to express the viscous part of the induced drag coefficient [41].

Most of the authors provide expressions of the Oswald factor in the form (7) proposed before. Others provide empirical solutions, following wind tunnel data [41].

In [42] the author provides an empirical diagram obtained from flight testing several modern and less modern aircraft. He proposed 1.05 and 0.007 for the values of Q and P respectively, that is:

$$e=\frac{1}{1.05+0.007\pi AR}$$

### 2.2. Energy and Propulsion System

The solar cells, energy storage blocks, propulsion group, payloads, and power electronics define the energy and propulsion systems architecture of a Solar UAV.

**Figure 3:**
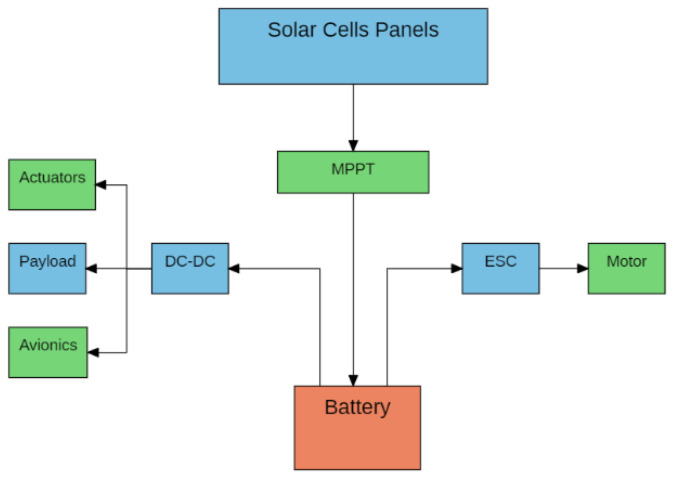
Basic electrical system of a Solar UAV.

The propulsion group comprises the motor, propeller, and motor controller, while the power electronics can use auxiliary systems that optimize power conversions, such as MPPT and DC/DC converters. Finally, we have the payload, actuators, and avionics of the aircraft [43]. Figure 3 shows the basic electrical system of a Solar UAV.

## 3. Sizing of Solar UAV

The sizing process of the aircraft is one of the initial steps from aeronautical design. Normally, it uses historical data and regression analysis from past projects to perform this estimation [44]. Once we create the conceptual design for the aircraft, it must ensure that the estimated weight is sufficiently accurate to meet mission requirements [45].

In designing a Solar UAV, new variables, such as a solar cells panel, make the process more complex and interactive. Furthermore, the addition of a solar cells panel to supply the energy demand directly influences the wing area. Therefore, it directly impacts the total weight of the aircraft and its energy demand. 

We should size a Solar UAV according to some design steps:

1. Set mission parameters: payload weight and power demand, flight speed, endurance, mission location, altitude of operation, solar cell and battery model and determine the aerodynamic profile, finding $C_{L}$, $C_{D0}\;$and e;
2. Adequate survey of the available solar resource at the mission location on the day of the year with less incidence of sunlight;
3. Assume a value for the area of the solar panel, calculate the solar energy collected. Based on the solar panel area, estimate the wing area and set the wing chord (c) based on the solar cell width. Obtain the wingspan and AR, based on wing chord and wing area. Calculate the lift and drag forces;
4. Assume a value for the total weight of the aircraft;
5. Calculate the required propulsion and total energy demand (power for mission profile) and compare with the value of solar energy collected in 3;
6. Sizing storage block and the total weight of aircraft and compare with the value in 4.

**Figure 4:**
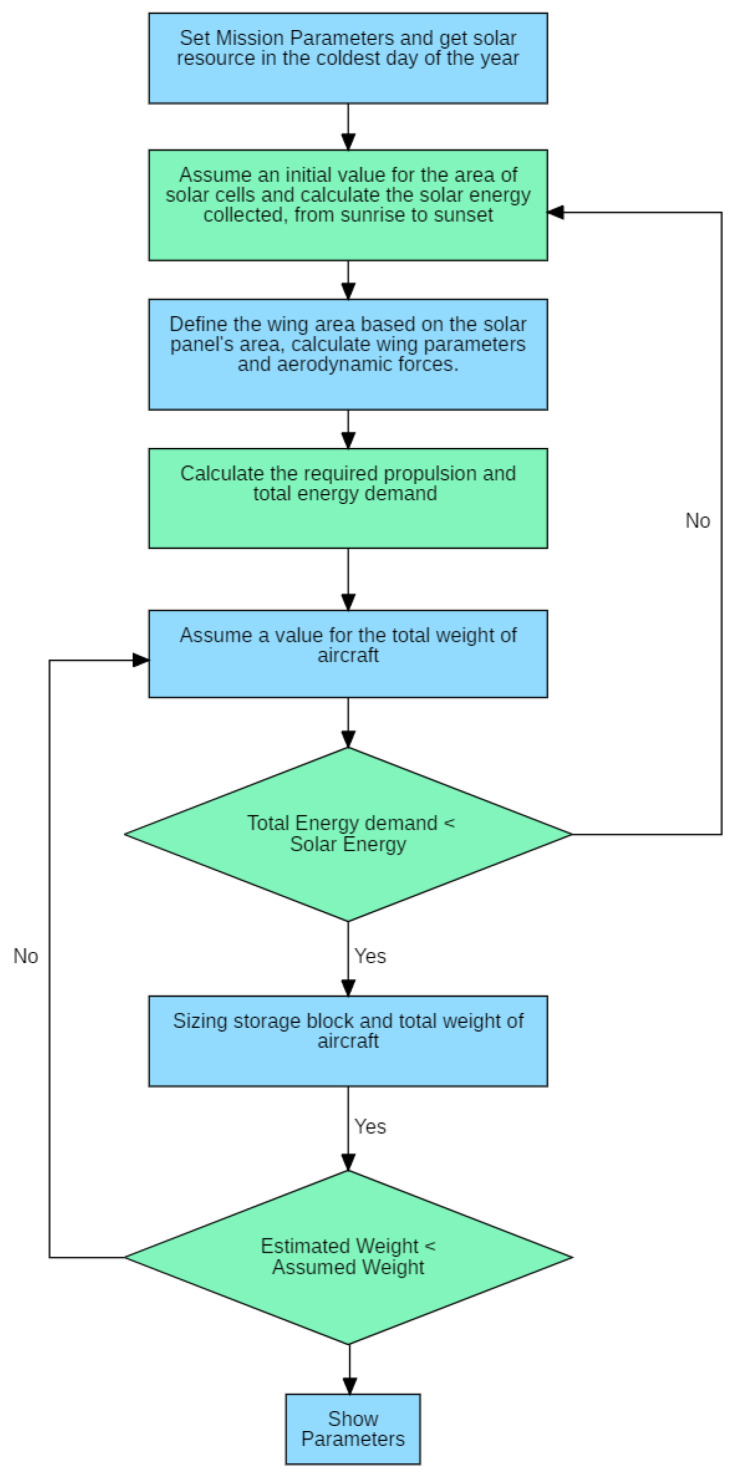
Flowchart of the UAV sizing process.

Iteration in steps 3 through 6 may be required several times. For example, in the sizing in step 6, the result for the total weight may require an increase in power for cruise flight since it requires a greater lift to support the heavier weight. Figure 4 shows a flowchart of the UAV sizing.

### 3.1. Mission Parameters (Step 1)

The definition of the operating altitude of the aircraft is one of the fundamental requirements of the mission. From this parameter, we obtain the absolute pressure and air density necessary for the calculation of lift and drag forces on the wing. In addition, the latitude and longitude of the mission are essential to define the solar resource available in the operating region.

The payload is the effective load that an aircraft carrier can perform on a specific mission. The size and mass of the payload and its electric power consumption are the major determinants of the aircraft’s layout, size, and overall mass.

The flight endurance of the aircraft may range from, for example, 1 h for a short-range surveillance system to over 24 h for a long-range surveillance system. The volume and mass of the power storage block determine the endurance. Thus, it matters in the definition of the total mass of the aircraft. 

The operational need of the mission determines the flight speed. This speed is a dominant factor in the propulsion configuration of the aircraft. 

Less weight and greater efficiency of the solar cell is an important aspect of performing aircraft. Furthermore, the addition of rigid solar cells on the aircraft might affect the shape of the UAV wing. Semi-flexible cells, such as the SunPower C60, are ideal, because of their low weight, high efficiency, and ease of assembly.

The specific energy density (Wh/kg) is the main parameter to consider when choosing the power storage block of a UAV, since any increase in weight requires increased propulsion power. Because of its high specific energy density, most of today’s solar aircraft, such as Sky-Sailor [32], use lithium-ion batteries, such as the NCR 18650B model. 

### 3.2. Solar Resource Assessment (Step 2)

This project stage seeks to quantify the incident global solar irradiation on the photovoltaic panel in a region.

For critical systems, such as those used in UAVs, it is necessary to size the system to operate on the day with the lowest incidence of solar radiation. If the system can operate under these conditions, it can operate on other days of the year.

Some parameters, such as sunshine duration and cloud cover, affect available solar radiation. This radiation is the determining factor in defining the aircraft’s wing area. There are several models to estimate the value of solar radiation. Some of them are empirical models which use mathematical formulae. There are also models based on atmospheric characteristics and satellite data such as ozone absorption, and Rayleigh scattering for forecasting solar radiation [46]. It is necessary to point out that the aircraft flight and mission profile will be accomplished according to the design of the aircraft assumptions. To consider general weather conditions for a specific aircraft project, there are necessary tests to feedback the design or match the site conditions to the flight or mission profile. A reasonable solution for general weather is to assume an empirical factor < 1 to multiply the calculated autonomy. The designer or user will obtain more realistic insight after evaluating results.

In this research, we used a model that considers the amount of sunlight available and the possibility of cloud cover. The first parameter found is the declination δ, and the angular position of the sun at the solar noon. To find the declination for a day *n* of the year, we use [47]:

(8) $$\delta =23.45\;\mathrm{sen}\left(360\;\frac{284+n}{365}\;\right),$$

where n is the day of the year, specified as a sequence, ranging from 1 for 1 January to 365 for 31 December. Thus, Equation (8) produces 365 values of declination δ, with a variation in the parameter *n*.

The next parameter is the hourly angle of the sunset $\omega_{s}$, obtained as a function of latitude ϕ and declination δ and calculated by:

(9) $$\omega_{s}=cos^{-1}(-\tan\phi \tan\delta ).$$

From $\omega_{s}$, we obtained the number of hours (*N*) with the availability of sun, according to [48]:

(10) $$N=\frac{2}{15}*\omega_{s}.$$

The declination values produce 365 values of $\omega_{s}$, in Equation (9) and *N* in Equation (10), the smallest of the values of *N* corresponds to the day of the year with the lowest incidence of solar radiation.

For altitudes less than 2.5 km, we estimated the total average daily extraterrestrial irradiance ($H_{O\;}$), available from the sun using [47]:

(11) $$H_{O\;}=\;\frac{24*1367\;}{\pi }\left(\;1+0.033\;\cos\frac{360N}{365}\right)*\left(\cos\phi \cos\delta \sin\omega_{s}+\frac{\pi \omega_{s}}{180}\sin\phi \sin\delta \right).$$

**Figure 5:**
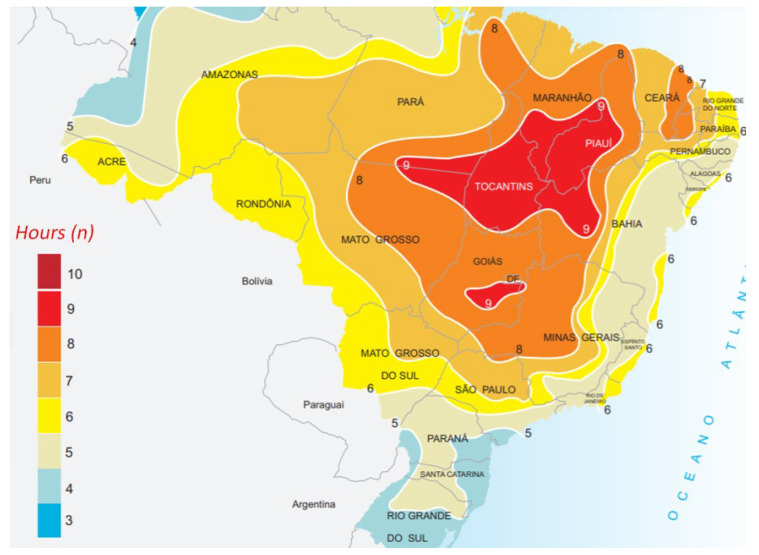
Monthly average daily insolation for the month of June, in Brazil, source: Adapted from [49].

We estimated the average daily, monthly radiation $\bar{H}\;$on a horizontal surface, considering the effects of the atmosphere, using [48]:

(12) $$\bar{H}=H_{O\;}*\left(0.16+0.87\frac{\bar{n}}{\bar{N}}-0.61{\left(\frac{\bar{n}}{\bar{N}}\right)}^{2}+0.34{\left(\frac{\bar{n}}{\bar{N}}\right)}^{3}\;\right),$$

where $\bar{N}$ is the daily monthly average of hours with the availability of sun, i.e., the average duration of the day in the month, calculated as the average of the number of hours N for each month of the year, $\bar{n}$ is the monthly average daily insolation (hours), available at many hundreds of weather stations in many countries. Figure 5 shows the value of $\bar{n}$ for the month of June, in Brazil.

**Figure 6:**
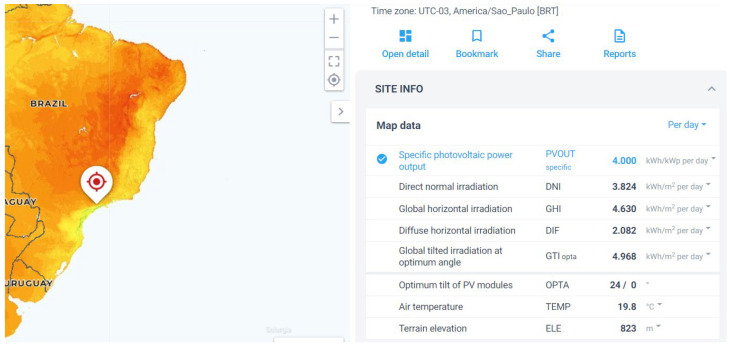
Solar irradiation data for the city of São Paulo, Brazil. Source: [50].

Here, we propose the use of a data source Global Solar Atlas [50] that considers the daily average global horizontal irradiation to obtain the value of $\bar{n}$. We divided the daily average global horizontal irradiation by the nominal solar energy power, i.e., $1000\;W\;/\;m^{2}$, to obtain $\bar{n}$. Figure 6 shows the daily average global horizontal irradiation in the city of São Paulo, Brazil.

Finally, we estimated the total hourly radiation $I_{b}$, per square meter, by [47]:

(13) $$I_{b}=\bar{H}*\left(\frac{\pi }{24}\left(c+d\cos\omega \right)\frac{\cos\;\omega -\cos\;\omega_{s}}{\sin\;\omega_{s}-\frac{\pi \omega_{s}}{180}\;\cos\omega_{s}}\right),$$

where *ω* is the hourly angle for each hour of the day (Hour), provided by:

(14) ω=(15Hour−180)π/180,

for radians, or for degrees using:

(15) ω=( Hour−12)∗15.

The constants c and d correspond to [47]:

$$c=0.409+0.5016\;\sin\left(\omega_{s}-60\right),d=0.6609-0.4767\;\sin\left(\omega_{s}-60\right).$$

We summed the radiation $I_{b}$ for each hour during the day to find the total solar energy in one day ($I_{T}$, per square meter).

### 3.3. Solar Energy Collected and Wing Parameters (Step 3)

Initially, we assumed a value for the area of the solar cells panel, and then, we calculated the wing area, considering the total area of the solar cells panel divided by a fill factor, that is:

(16) $$S=\frac{Panel_{area}}{Fill_{Factor}}.$$

The fill factor is empirical and depends on the solar cell model used. For example, on a solar panel, we consider the effective area of the solar cells panel mounted on that panel.

Solar energy is obtained according to [33,51] by:

(17) $$P_{solar}=I_{T\;}*\;A_{C\;}*\eta_{sc}\;*\;\eta_{mppt}*\;\eta_{cbr},$$

where $I_{T}$ is the total solar radiation incident on the day of the year with the lowest incidence of solar radiation, $A_{C}$ is the area of the solar cells, $\eta_{sc}$ and $\eta_{mppt}$ are the solar cell and maximum power point tracker efficiencies, respectively. The parameter $\eta_{cbr}$ accounts for solar module level losses mainly caused by the camber of the cell arrangement. These losses are typically in the order of 10% [32].

#### Aerodynamic Profile

The aerodynamic profile defines lift and drag coefficients. Therefore, a database containing the detailed characteristics of various aerodynamic profiles would be ideal for solar UAV designers. Airfoil tools [52] is an online database that contains detailed information on thousands of aerodynamic profiles. This information can define the values of the wing’s lift, drag, and profile drag coefficients.

### 3.4. Power for Mission Profile (Steps 4 and 5)

The first step to determine the total required power for a UAV mission is to define the flight mission segments or mission profile. The mission profile can be divided into takeoff, climb, cruise or level flight, descent, and landing.

#### 3.4.1. Power for Level Flight

In level flight, lift and weight forces are equal, as well as drag and thrust forces. So, we have two equations [53]:

(18) L=W,  D=T.

The power required for level flight is provided by [30]:

(19) $$P_{level}=\left(\frac{C_{D}}{C_{L}{}^{\frac{3}{2}}}\right)\sqrt{\frac{2{\left(m_{tot}\;g\right)}^{3}}{\rho \left(h\right)A_{wing}}}\;,$$

where, $m_{tot}$ is the total airplane mass, g is the gravity constant, $A_{wing}$ is the wing area, and ρ(h) is the altitude-dependent air density.

Considering the efficiency of the different components of the propulsion system, the total power in level flight is determined by:

(20) $$P_{level}=\frac{P_{level\;}}{\eta_{prop}}+P_{av}+P_{pld},$$

where $\eta_{prop}$ includes propeller, gearbox, motor, and motor controller efficiency, $P_{av}$ is the avionics power and $P_{pld}$ is the payload power. 

#### 3.4.2. Power for Climb Flight

For the estimation of the power in climb flight, we choose a reasonable climbing angle $\alpha_{cl}$ (between 20° for hand-launched models and 10 ° for aircraft with very large wings) at climbing speed $v_{cl}$ [33].

In cruise flight, the thrust force is the correspondent drag force, but for climb flight, the estimation uses the constant rate of climb. Here, the physical difference concerning level flight is that the thrust not only acts to overcome drag, but on climb flights it also supports a weight component.

Therefore, the required thrust for climbing is provided by [54]:

(21) $$T=D+W\;\sin\alpha_{cl}.$$

And the net power during the climb flight is:

(22) $$P_{net}=Tv_{cl}=Dv_{cl}+Wv_{cl}\;\sin\alpha_{cl},$$

where $v_{cl}$ is climbing speed, provided as a function of stall speed.

The stall speed is the minimum speed at which an aircraft can safely lift off from the ground. For safety, it requires the climbing speed to be greater than the stall speed. Considering a value 20% higher, we have [55]:

(23) $$v_{cl}=1.2V_{stall}=1.2\sqrt{\frac{2W}{\rho SCl_{max}}},$$

where $Cl_{max}$ is the maximum lift coefficient of the aircraft.

It is necessary to multiply the net power during the climb by the time to climb to obtain the power consumed during the climbing flight, that is:

(24) $$P_{climb}=\frac{P_{net\;}}{\eta_{prop}}*time\;to\;climb.$$

We obtained the time to climb from the rate of climb, and the vertical speed of the plane. From [40] comes the expression:

(25) $$R/C=\frac{dh}{dt}=v_{cl}\cdot \sin\alpha_{cl}=v_{cl}\;\cdot \;\frac{T-D}{W}\;,$$

where *h* stands for altitude, *T* for thrust, *D* for drag, *W* for weight, and $\alpha_{cl}$ for the climb angle. 

Considering T−D as the excess power, Equation (25) becomes

(26) $$\frac{R}{C}=v_{cl}\;\cdot \;\frac{Excess\;Power}{W}.$$

Speed is simply the temporal rate of change in the distance; in this case, the distance is the altitude h, therefore:

(27) $$dt=\frac{dh}{R/C},$$

where dt is a small increment of time required for the small increment dh in altitude. Therefore, the time to climb from an altitude h1 to another h2 is obtained by integrating Equation (27) [54]:

(28) $$t=\int_{h1}^{h2}\frac{dh}{R/C}.$$

For steady climbing flight lift becomes

(29) $$L=W\;\cos\alpha_{cl}.$$

As the altitude increases, L (and therefore $C_{L}$) decrease, so the induced drag is also less. As a result, the total drag for the climbing flight is less for the level flight with the same speed [54].

Rearranging Equation (3) to obtain $C_{L}$

(30) $$C_{L}=\frac{W\;\cos\theta \;}{\frac{1}{2}\;\cdot \rho \cdot v^{2}\cdot S}.$$

From the new value of $C_{L}$, we calculate $C_{Di}$ from Equation (6), Cd from Equation (5), and D from Equation (4).

With the decrease in the total drag of the aircraft, we have a decrease in excess power, decreasing the R/C according to Equation (26).

**Figure 7:**
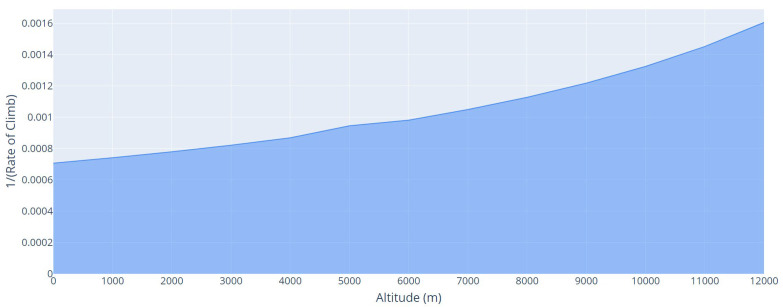
${\left(R/C\right)}^{-1}$ versus altitude curve. Takeoff altitude = 0 m, cruise altitude = 12,000 m.

We obtained the time to climb from different values of *R*/*C*, obtained by the distance traveled between the takeoff altitude and the cruise altitude. To calculate the time of climb graphically, we plotted ${R/C}^{-1}$ versus altitude values on a graph, as shown in Figure 7. The area under the curve from takeoff altitude to cruise altitude is the time to climb [54].

**Figure 8:**
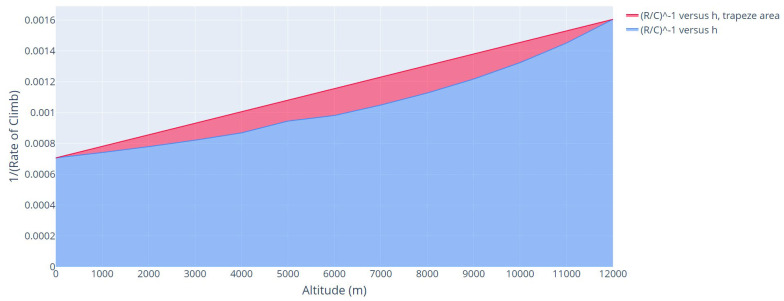
${\left(R/C\right)}^{-1}$ versus altitude, trapeze area. Takeoff altitude = 0 m, cruise altitude = 12,000 m.

For safety, only the *R*/*C* values of the initial and final altitude were plotted, bringing the area of the ${\left(R/C\right)}^{-1}$ versus altitude graph closer to the area of a trapeze, as shown in the red area of Figure 8. Thus, the area considered in the time to climb estimation is the sum of the blue area and the red area. Finally, the time to climb is multiplied by 2, considering the descending flight as equal to the climbing flight, that is:

(31) $$t_{climb}=2t.$$

Considering aircraft that take off from a runway, an important parameter is the distance required for takeoff and landing, and the total power required for this stage. The same aircraft may require different runway distances to operate, depending on some parameters, such as air temperature, altitude, wind velocity, and runway declivity. For an aircraft to lift off the ground, the wings must produce a lift force greater than the aircraft’s weight. Four variables are essential to produce such force, airspeed, air density, wing area, and lift coefficient [56]. The takeoff and landing distances are provided by [40]:

(32) $$S_{takeoff}=\frac{1.44W^{2}}{g\;\rho \;S\;C_{lmax\;}\left\{T-\left[D+\mu \;\left(W-L\right)\right]\right\}},$$

(33) $$S_{landing}=\frac{1.69W^{2}}{g\;\rho \;S\;C_{lmax}\;\left[D+\mu \;\left(W-L\right)\right]},$$

where μ is the friction coefficient between the aircraft wheels and the ground. This coefficient can vary from 0.02 for paved runways to 0.1 for grass runways. In [40] a list of the friction coefficient with the type of runway material is provided.

As the values of drag and lift forces change as the speed increases, the calculation of Equations (31) and (32) become very complex. As a way of simplifying the solution, the author in [57] suggests using 70% of the takeoff speed for an average flight velocity. Equations (3) and (4) calculate the values of L and D, considering $v=0.7v_{cl}$, therefore:

$$L=\frac{1}{2}\;\rho \;{\left(0.7v_{cl}\right)}^{2}\;S\;C_{L},$$

$$D=\frac{1}{2}\;\rho \;{\left(0.7v_{cl}\right)}^{2}\;S\;\left(C_{Do}+\phi \;C_{Di}\right),$$

where ϕ is the ground effect, the reduced aerodynamic drag that an aircraft’s wings generate when they are close to a fixed surface. Its value is:

(34) $$\phi =\frac{{\left(16\;\;h/b\right)}^{2}}{1+{\left(16\;h/b\right)}^{2}},$$

where h is the height between the wing and the ground, and b is the wingspan.

The time required to travel the runway during takeoff is

(35) $$t_{takeoff}=\frac{S_{takeoff}}{0.7v_{cl}},$$

and, for landing:

(36) $$t_{landing}=\frac{S_{landing}}{0.7v_{cl}}.$$

Finally, the total time to climb is the sum of the time to climb, time to takeoff, and time to landing, that is:

(37) $$time\;to\;climb=t_{climb}+t_{takeoff}+t_{landing},$$

which can now be used in Equation (24).

### 3.5. Weight Prediction (Step 6)

Weight prediction is an essential part of the aircraft design process [44]. It affects the cost and performance characteristics of an aircraft [58]. We can categorize weight prediction into finite element, empirical, and semi-empirical approaches. Finite element weight prediction provides accurate and reliable estimates of airframe mass but they are problem specific; not readily generalized and would increase the computational cost of the process. Empirical methods estimate the mass of the aircraft’s main component group, such as a wing or fuselage, using empirical equations that combine geometric parameters, aircraft design speeds, load factor, and statistically derived coefficients. In this approach, we estimated the weight of the individual components of aircraft with the help of curve fitting methods. The sum of these weights is the overall empty weight, or gross takeoff weight. Empirical methods are easier to implement and more efficient than the finite element approach. However, they are of low fidelity. Semi-empirical methods comprise analytically derived equations corrected with the statistical correlation from historical data [59]. The semi-empirical method is the most suitable weight prediction method for the conceptual design synthesis of aircraft. This is because they are easy to implement, as well as sufficiently accurate for the conceptual design phase [60]. Therefore, this is the method chosen for weight prediction in this research.

Weight prediction of Solar UAV starts with the guess of gross takeoff weight, which considers the weights of the structure, propulsion group, solar cells, storage block, and payload, based on the following weight breakdown equation:

(38) $$W_{Total}=W_{fixed}+W_{solarcells}+W_{energystorage}+W_{structure}+W_{propulsion}.$$

#### 3.5.1. Fixed Weights

Some fixed weights do not depend on the sizing of other parts of the aircraft, such as the payload, which is a mission parameter defined at the beginning of the project. Besides the payload, we can consider avionics, such as autopilot, and micro-controlled systems, such as a Raspberry Pi board. Thus, fixed weight is:

(39) $$W_{fixed}=W_{payload}+W_{avionics}.$$

#### 3.5.2. Weight Prediction of the Solar Cells

Using commercially available solar cells simplifies the mass prediction of this step, since the manufacturer provides the nominal weight values. The mass of the solar panel is:

(40) $$W_{solarcells}=\frac{Panel_{area}}{Cell_{area}}*cell_{weight\;}*g*k,$$

where $Cell_{area}$ and $cell_{weight\;}$ are the nominal values of the area and weight of the solar cell used, g is the gravity constant and k is a constant (k > 1) to represent the weight portion of the solar cells panel encapsulation.

#### 3.5.3. Weight Prediction of the Energy Storage Block

We based the weight of the energy storage block on the energy stored for the endurance of the Solar UAV. The energy stored is:

(41) $$Energy_{bat}=\frac{1}{\eta_{bat}}\left(P_{level}*\;T_{endurance}+P_{climb}\right),$$

where $\;P_{level}\;$and $P_{climb}$ were calculated by Equations (20) and (24), respectively, but now it must consider the efficiency of the energy storage block. $T_{endurance}$ is defined as flight endurance.

From Equation (41), we can divide the energy stored in the battery by the specific energy density of the battery (Wh/kg) that is chosen during the definition of the project and multiply by gravity, that is:

(42) $$W_{energystorage}=\frac{Energy_{bat}}{Density_{bat}}*g.$$

In [32], a 10% increase in the weight of the energy storage block, propulsion group, and solar cells is proposed due to the weight of the wiring, transformers, controllers, etc.

#### 3.5.4. Weight Prediction of the Aircraft Structure

To estimate the weight of the aircraft structure, the author in [32] proposes a new empirical model, validated using a database containing the parameters of 415 aircraft of various dimensions, divided into 92 radio-controlled unmanned models and 323 manned aircrafts. The following equation is used to estimate the weight of the aircraft structure:

(43) $$W_{structure}=g\;K\;S^{x1}AR^{x2},$$

where g is the gravity constant, S is the wing area, AR is the aspect ratio. *K*, *x*1, and *x*2 are constants that vary according to the construction quality of the aircraft and where obtained from a table in [32].

#### 3.5.5. Weight Prediction of the Propulsion Group

Aircraft launch conditions have a significant influence on the power to mass ratio of the propulsion group elements. Airplanes that take off from a runway smoothly increase their speed until takeoff, so the difference between the takeoff power and level flight power is low. Aircraft launched manually need to increase their speed and gain altitude quickly. In these cases, the engine must provide a starting power much greater than the power supplied in level flight. This over-sizing is necessary for takeoff, but it also helps to fly in headwinds and turbulence [32].

Therefore, for the contribution of the propulsion group in the total weight of the aircraft, we use the following formula, proposed by [32], based on the over-sizing for hand-launched aircraft, which already considers the contribution of the motor, motor controller, and propeller:

(44) $$W_{propulsion}=0.008*\;P_{req\;}*g\;.$$

The estimated total weight should be compared with the weight assumed initially in the calculation of propulsion power ($m_{tot}$ in Equation (19)), which in this case corresponds to the lift force calculated in Equation (3). If the estimated weight is greater than the assumed weight, a new value is assumed for the initial total weight and the cruise propulsion power is recalculated.

## 4. Results and Discussion

### 4.1. Solar Resource Assessment Model

Many studies related to Solar UAV [32,34,61] use the model proposed by [47] to estimate solar radiation in a given region and period of the year. However, this model uses the following equation, instead of Equation (12), to estimate the monthly average daily solar radiation on a horizontal surface:

(45) $$\bar{H}=H_{O\;}*\left(a+b\frac{\bar{n}}{N}\right).$$

Equation (45) employs two empirical constants a and b that explain the local climate, such as tropical forest, desert, steppe or semiarid. The work in [47] provides a list of varied climates around the globe, with the values of constants a and b for some regions. In addition, in the equation, the value of *n* is obtained from meteorological data for the region and is also provided by the user.

As one aim of the proposed methodology is to simplify the number of parameters provided by the user, we employ Equation (12), which eliminates the use of the constants *a* and *b*, and we can obtain the value of *n* at the time of simulation, through a web request from the website global solar atlas.

Table 1 shows a comparison between the previous model and the proposed model. We obtained the value of $\bar{n}$ from [49] and the values of the constants a and b were 0.26 and 0.5, respectively, an approximation of the values provided by [47] for the city of São José do Campos. The day of the year with the lowest incidence of solar irradiation was June 22.

As one can see in Table 1 the solar radiation of both models is very close, but the proposed model matches the aim of this extended methodology, to simplify the number of parameters provided by the user.

### 4.2. Parameters Sensitivity and Constraints Analysis

To show the interrelation between the several parameters a multiparameter analysis was performed. Such analysis evidence which designs parameters is more sensitive in the aircraft sizing. The simulations demonstrate the critical parameters of the aircraft mission, identified as the main optimization objectives. To develop the simulations a basic condition with constant parameters was adopted.

Table 2 shows the mission parameters that remained fixed during the variation in the other parameters.

The proposed algorithm has two main functions: finding the minimum wingspan for constructing the embedded solar panel, producing enough energy to carry out the mission, and optimizing the wingspan to obtain the best relation between aircraft mass and power for cruise flight. Thus, it is desirable that the mass/power ratio is as high as possible. These are the maximum values of the curves, highlighted in these simulations.

**Figure 9:**
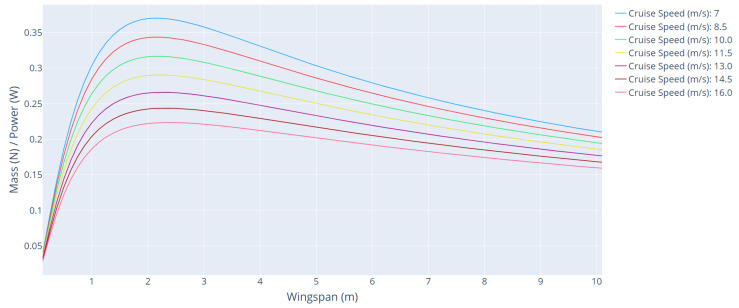
Mass/power ratio as a function of wingspan for different cruising speeds.

Figure 9 shows the curves of the mass/power ratio as a function of wingspan for different cruising speeds. Table 3 shows the main parameters of the aircraft for each speed range. 

From this analysis, it is possible to verify the great sensitivity of cruise speed in aircraft sizing. This is expected due to the quadratic relationship of speed in the drag force generation (Equation (4)). The higher the speed, the more power the aircraft needs to produce to overcome the drag forces. Thus, this value must be minimized.

The aircraft’s mission will usually require a payload with fixed mass; however, it is important to optimize this parameter, since the extra weight in the payload represents greater flexibility in the aircraft’s assembly, where additional parts and screws may be needed. Weather characteristics can add weight to the aircraft, such as ice on the wing. Therefore, the payload mass should be maximized.

**Figure 10:**
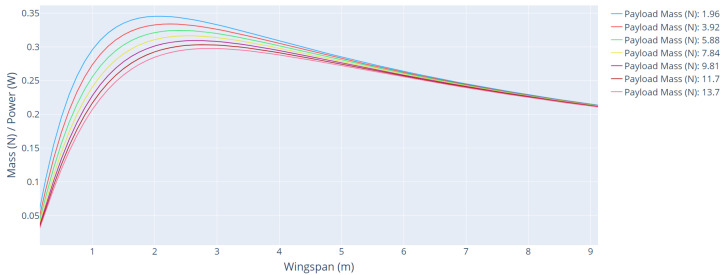
Mass/power ratio as a function of wingspan for different payload mass.

Figure 10 shows the curves of the mass/power ratio as a function of wingspan for different payload mass. Table 4 shows the main parameters of the aircraft for each payload mass range.

The payload mass values directly impact the total mass of the aircraft, and therefore the cruise power and climb power needed to carry that mass. However, the effect is much smoother than the cruise speed impact.

The longer the aircraft’s flight duration, the greater the area it can cover in carrying out the mission before needing to land to recharge its batteries. Therefore, flight endurance must be maximized.

**Figure 11:**
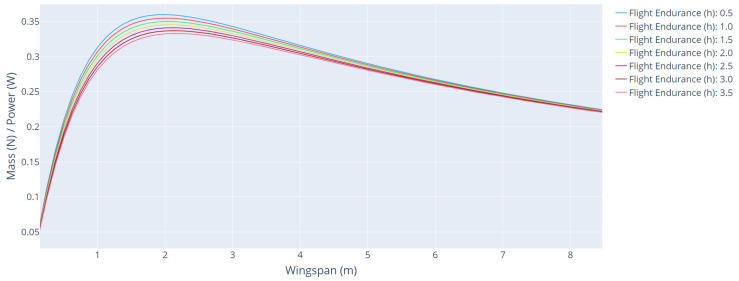
Mass/power ratio as a function of wingspan for different flight endurance.

Figure 11 shows the curves of the mass/power ratio as a function of wingspan for different flight endurance. Table 5 shows the main parameters of the aircraft for each flight endurance range. 

The mass/power ratio curves as a function of wingspan for different flight endurance showed the smallest amplitude compared with the other curves, proving that this factor is the least sensitive in optimization.

**Figure 12:**
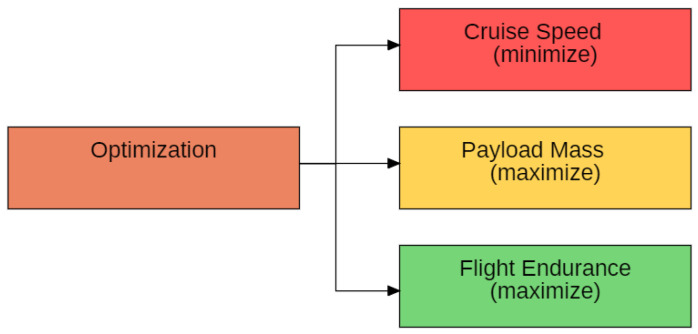
Parameters to be optimized by the algorithm.

Figure 12 shows the parameters to be optimized according to design sensitivity.

### 4.3. Differences between Mission Profiles

In this section, we performed an energy profile and weight comparison between an aircraft with a mission that considers climb power and an aircraft that does not.

**Figure 13:**
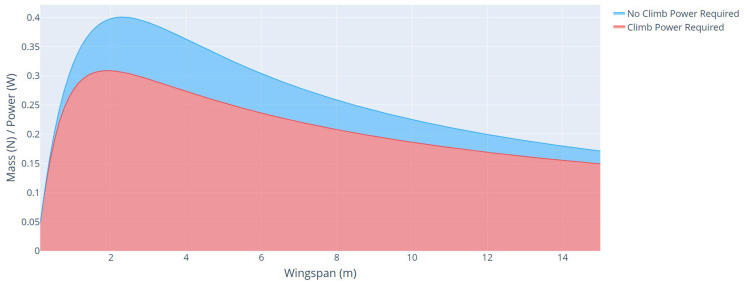
Mass/power ratio as a function of wingspan for aircraft with different mission profiles.

Figure 13 shows the curves of the mass/power ratio as a function of wingspan for an aircraft with a mission that considers climb power and an aircraft that does not. Table 6 shows the main parameters of each aircraft.

**Figure 14:**
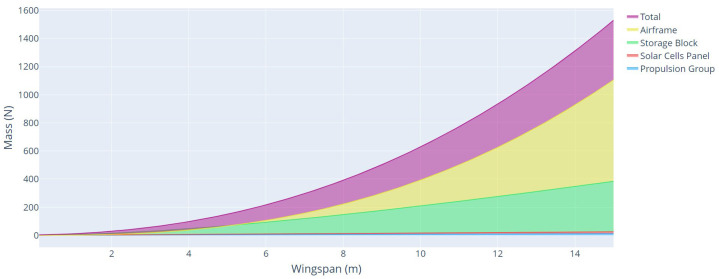
Aircraft mass as a function of wingspan with climbing power.

**Figure 15:**
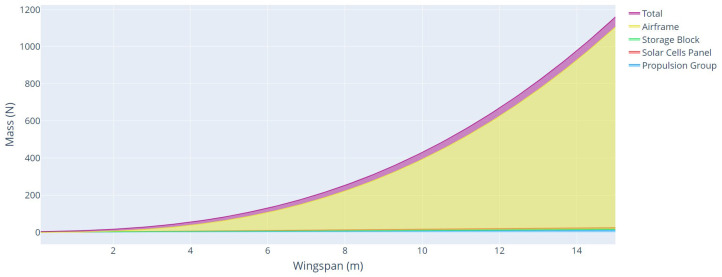
Aircraft mass as a function of wingspan without climbing power.

The last parameters analyzed were the total weight and weight of each aircraft component, as a function of the wingspan, for each type of mission profile, with climbing power, and without climbing power, as shown in Figure 14 and Figure 15, respectively.

From these analyses, it is possible to verify the great impact that the climb power represents on the UAV solar project. For a mission in the same region and with the same characteristics of speed, range, mass, and payload power, the power for the cruise flight represented an increase of about 191% compared with an aircraft that disregards the climb power in the mission profile. This increase is mainly because of the power consumed by the aircraft during the climb and the addition of weight in the battery to store this power.

### 4.4. Python Framework

We developed a free opensource framework for sizing Solar UAVs, using the Python language and the open-source libraries Folium [62], Numpy [63], Matplotlib [64], and PyQt [65]. The main aim of this framework is to facilitate the designer’s interaction in the main stages of the Solar UAV project, simulating the aircraft’s output parameters, such as wingspan, total mass, and propulsion energy, from the mission parameters such as altitude, flying velocity, payload mass, payload power, and latitude. This framework will be available soon, in the following repository [66].

To test the developed Framework, we proposed a surveillance mission along the Paraibuna river, at latitude −23.396648 and longitude −45.598283. Table 7 shows the main parameters of the mission.

**Figure 16:**
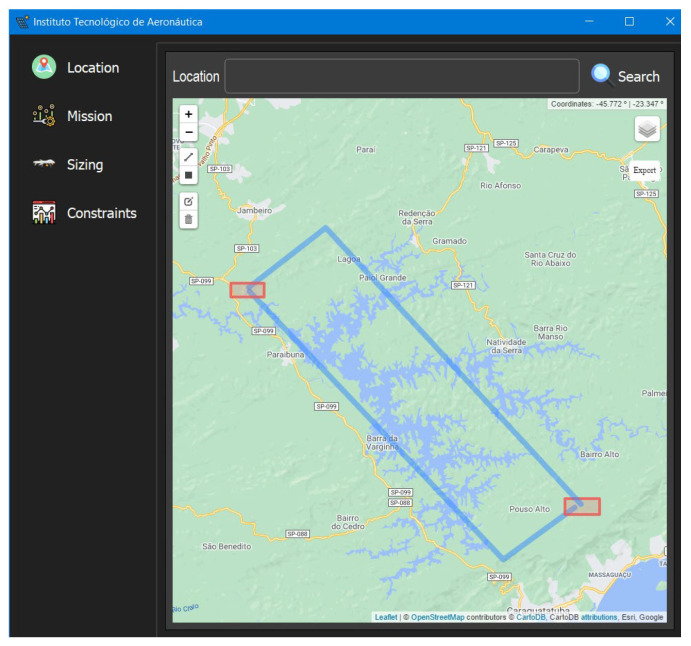
Definition of the place of operation.

The first step of the design is the definition of the place of operation of the aircraft, as shown in Figure 16. On the map, the user draws the trajectory of the aircraft. The blue lines represent the route traveled and the red rectangles represent the aircraft’s landing and takeoff locations.

**Figure 17:**
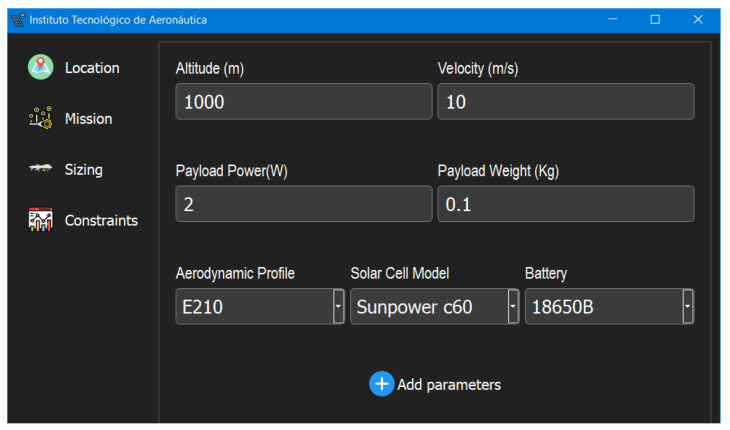
Fields for the mission parameters.

In the next step, the fields for the mission parameters are filled, as shown in Figure 17.

Selecting the aerodynamic profile, the solar cell model and the battery model, the framework finds, from a database, the lift and drag coefficient, the density and the efficiency of solar cell, and density and efficiency of the battery. The user can add new components directly in the database of the framework.

**Figure 18:**
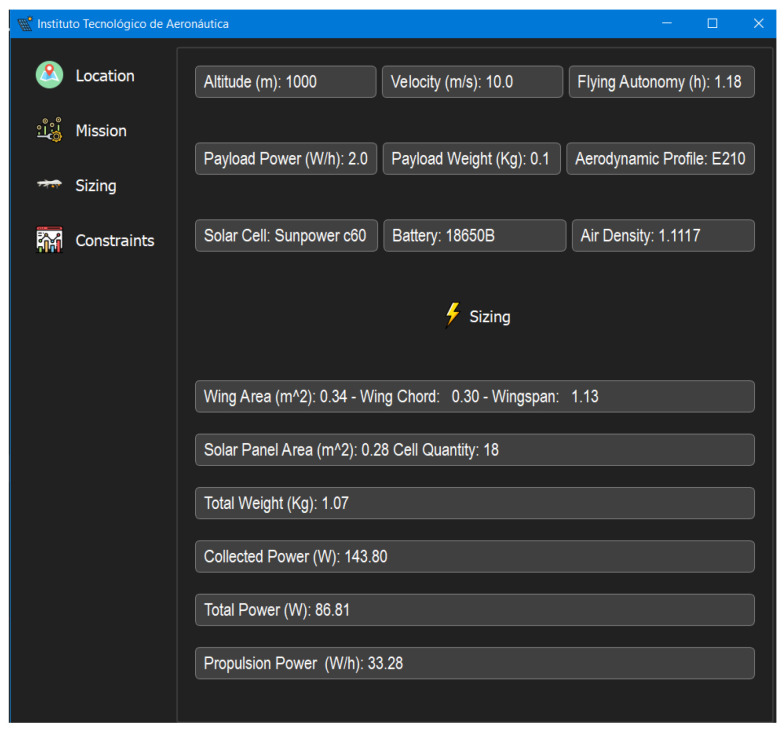
UAV Sizing results.

The last stage of the framework operation is the sizing itself, as shown in Figure 18, where it is possible to visualize the aircraft output parameters, as shown in Table 8. Table 9 shows the aircraft optimized output parameters.

### 4.5. Contributions of the Study 

The contributions of this study are summarized as follows:

- A complete model for estimating solar radiation on the day of the year with less incidence of sunlight. There is no longer a need for specific data provided by meteorological stations;
- The Oswald efficiency factor is now estimated before we should provide this value to estimate the induced drag of the aircraft;
- A complete model to estimate the power needed for the flight, including the different flight phases (takeoff, climb, descent and landing), including the type of runway material;
- Changes to some details in the weight prediction model to account for commercially available solar cells and batteries;
- Regarding the framework, the use of Python language, instead of Matlab, with only open-source libraries, the definition of the aircraft route in an interactive map, detailed analysis of design constraints, and optimal point analysis of sizing measures, varying the mission parameters.

The above contributions are beyond the results achieved early in [34,35]. It is important to highlight that the proposed methodology aiming a mission with limited flight time, instead of a long-range or perpetual flight, as proposed in [30,31,32,33].

## 5. Conclusions

After initial considerations and information about Solar UAV designs, an extended methodology for the conceptual design of solar airplanes was presented and compared with original studies.

The proposed methodology enables the evaluation of the role of several parameters to find the optimal relationship between the total weight of the aircraft and the power needed to carry out the cruise and climbing flight. This methodology uses mathematical models that relate the sizing of all aircraft elements, from the concepts of energy and mass balances during the year’s day with less incidence of sunlight.

Comparing the solar radiation information data for the proposed methodology with early works of solar models reveals the data are consistent, even with the simplification adopted, which fulfills the aim of the new solar model, to reduce the number of parameters provided by the user.

We performed a multi parameters analysis and showed the interrelation between mass/power ratio, cruise speed, payload mass, and flight endurance. Such analysis evidence, which designs parameters, is more sensitive in the aircraft sizing and obtained the highest sensitivity of the mass/power ratio with the cruise speed.

The methodology enables the authors to develop a framework with a graphical user interface, using Python to simplify user interaction. We show the framework usability and output characteristics after sizing and optimizing a Solar UAV’s output parameters from a proposed mission profile and constraints according to the initial discussion and pointed aims.

To demand a small number of entries and data by the user, and taking into account the takeoff, climb, and landing energies, the methodology proves to be adequate for obtaining more realistic results in this type of aircraft, bring new results, and can be considered as a helpful tool for Solar UAV developments.

## Figures and Tables

**Table 1:** Comparison between different models to estimate solar radiation.

Model	a	b	$\bar{n}$	$\mathrm{Solar}\;\mathrm{Irradiation}\;\left(kwh/m^{2}\right)$
Previous Model	0.26	0.5	5	3.073
Proposed Model	-	-	5	2.989

**Table 2:** Fixed mission parameters of the simulation.

Climbing distance (m)	300
Solar energy available (W)	2989
Payload power (W)	2

**Table 3:** Main parameters of the aircraft for each cruise speed range.

Cruise Speed (m/s)	Wingspan (m)	Cruise Power (W)	Climb Power (W)	Mass/Power
7	2.00	47.357	95.98	0.3832
8.5	2.00	60.86	141.64	0.3547
10	2.00	79.93	196.23	0.3256
11.5	2.12	116.23	289.83	0.2976
13	2.12	154.25	370.81	0.2716
14.5	2.25	221.83	512.01	0.2479
16	2.25	290.92	624.33	0.2268

**Table 4:** Main parameters of the aircraft for each payload mass range.

Payload Mass (N)	Wingspan (m)	Cruise Power (W)	Climb Power (W)	Mass/Power
1.96	2.12	72.85	158.02	0.3453
3.92	2.25	88.34	175.18	0.3339
5.88	2.37	104.73	193.08	0.3244
7.84	2.50	122.01	211.71	0.3163
9.81	2.62	140.17	231.06	0.3093
11.7	2.75	159.24	251.11	0.3031
13.7	2.75	168.92	251.11	0.2973

**Table 5:** Main parameters of the aircraft for each flight endurance range.

Endurance (h)	Wingspan (m)	Cruise Power (W)	Climb Power (W)	Mass/Power
0.5	2.00	58.17	141.64	0.3597
1.0	2.00	60.86	141.64	0.3547
1.5	2.00	63.60	141.64	0.3499
2.0	2.12	72.85	158.02	0.3452
2.5	2.12	69.20	158.02	0.3408
3.0	2.12	75.80	158.02	0.3365
3.5	2.12	74.96	158.02	0.3323

**Table 6:** The main parameters for aircrafts with different mission profiles.

Aircraft Type	Wingspan (m)	Cruise Power (W)	Climb Power (W)	Mass/Power
No Climb Power	2.25	49.32	0	0.4005
Climb Power	2.00	94.63	141.64	0.3086

**Table 7:** Mission parameters for proposed mission.

Altitude (m)	1000
Flight Velocity (m/s)	10
Solar Energy Available (W)	2989
Payload Power (W)	2.0
Payload Weight (Kg)	0.1
Aerodynamic Profile	E210
Battery Model	18650B
Solar Cell Model	Sunpower C60

**Table 8:** Aircraft output parameters.

Wing Area (m)	0.34
Wing Chord (m)	0.3
Wingspan (m)	1.13
Solar Panel Area (m)	0.28
Solar Cell Quantity	18
Total Weight (Kg)	1.10
Collected Power (W)	143.8
Total Consumed Power (W)	86.81
Propulsion Power (W)	33.28
Flight Velocity (m/s)	10
Payload Weight (Kg)	0.1
Flight Endurance (h)	1.18

**Table 9:** Aircraft optimized output parameters.

Wing Area (m)	0.6
Wing Chord (m)	0.3
Wingspan (m)	2.00
Solar Panel Area (m)	0.49
Solar Cell Quantity	32
Total Weight (Kg)	1.10
Collected Power (W)	276.18
Total Consumed Power (W)	237.04
Propulsion Power (W)	63.6
Flight Velocity (m/s)	8.5
Payload Weight (Kg)	0.2
Flight Endurance (h)	1.5
